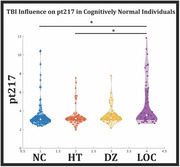# Exposure to TBI increases the susceptibility to tau phosphorylation at pT217

**DOI:** 10.1002/alz70856_104606

**Published:** 2025-12-26

**Authors:** Jeremy F. Strain, Chia‐Ling Phuah, Yingxin He, Owen Guo, Kyle B. Womack, Raquel C. Gardner, Tammie L.S. Benzinger, Suzanne E. Schindler, John C. Morris, Randall J. Bateman, Nicolas R. Barthélemy

**Affiliations:** ^1^ Washington University in St. Louis School of Medicine, St. Louis, MO, USA; ^2^ Dignity Health dba Barrow Neurological Institute/St. Joseph's Hospital & Medical Center, Phoenix, AZ, USA; ^3^ Washington University School of Medicine, St. Louis, MO, USA; ^4^ Sheba Medical Center, Joseph Sagol Neuroscience Center, Ramat Gan, Israel; ^5^ Hope Center for Neurological Disorders, Washington University School of Medicine, St. Louis, MO, USA; ^6^ Mallinckrodt Institute of Radiology, Washington University School of Medicine, St. Louis, MO, USA; ^7^ Knight Alzheimer Disease Research Center, Washington University School of Medicine, St. Louis, MO, USA; ^8^ Department of Neurology, Washington University School of Medicine, St. Louis, MO, USA; ^9^ The Tracy Family SILQ Center, Saint Louis, MO, USA; ^10^ The Charles F. and Joanne Knight Alzheimer Disease Research Center, St. Louis, MO, USA; ^11^ Washington University in St. Louis, St. Louis, MO, USA; ^12^ The Tracy Family SILQ Center, St. Louis, MO, USA; ^13^ Hope Center for Neurological Disorders, St. Louis, MO, USA; ^14^ Department of Neurology, Washington University in St. Louis School of Medicine, St. Louis, MO, USA; ^15^ SILQ Center for Neurodegenerative Biology, St. Louis, MO, USA

## Abstract

**Background:**

Alzheimer Disease (AD) is the most prevalent form of dementia and Traumatic brain injury (TBI) significantly increases the risk of developing dementia. Both AD and TBI are categorized as 3R+4R tauopathies but influence of TBI exposure on AD‐like tau phosphorylation in biofluids remains unclear. *p*‐tau217, has gained traction as an early indicator of tau abnormality in response to amyloid deposition in AD. We evaluated whether CSF tau phosphorylation, amyloid deposition, tau aggregation, and neurodegeneration were elevated in cognitively normal individuals with TBI history compared to individuals without TBI.

**Method:**

We analyzed CSF *p*‐tau measures in 242 cognitively normal individuals with TBI history quantified with OSU questionnaire. TBI history was ranked by symptom severity: no reported head trauma or TBI (NC), head trauma but no TBI (HT), TBI incident with dazing or confusion for >30 minutes (DZ), and lost consciousness (LOC) for >30 minutes due to the TBI incident. Tau phosphorylation occupancy at T181, T205 and T217 sites was quantified using Mass Spectrometry. PET SUVR summary metrics of amyloid and tau burden were quantified, and summarized neurodegeneration was estimated in AD specific gray matter regions. We constructed statistical linear models based on our primary hypothesis to investigate the relationship between TBI presence/severity at predicting %p‐tau217 and other representatives of the ATN framework.

**Result:**

This cohort comprised of 52 NC, 42 HT, 23 DZ, and 34 LOC. TBI severity classification did significantly predict pt217 (*p* = 0.0007) independent of age and sex. Non‐parametric tests revealed the LOC group had the greatest %p‐tau217 phosphorylation compared to NC (*p* = 0.0026) and HT (*p* = 0.011) but not the DZ group (*p* = 0.15). This effect was not observed for the other tau phosphorylated sites. PET and MRI measures revealed that TBI severity significantly predicted PET amyloid (*p* = 0.0368) with the LOC group exhibiting the greatest amyloid accumulation. TBI severity did not predict PET tau or neurodegeneration in this asymptomatic cohort.

**Conclusion:**

These data demonstrate an association between prior exposure to a severe TBI event with elevated amyloid deposition and selective %p‐tau217, suggesting an increasing vulnerability to AD progression, but does not appear to coincide with accelerated neurodegeneration in asymptomatic individuals.